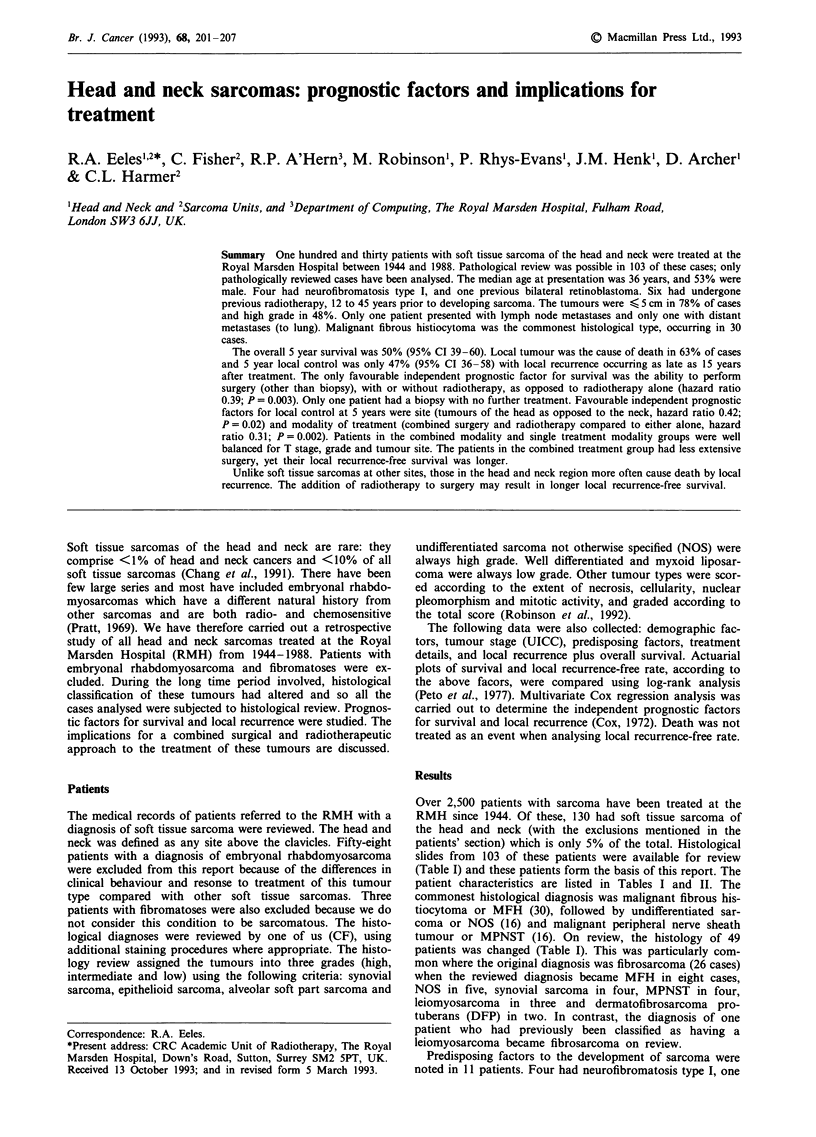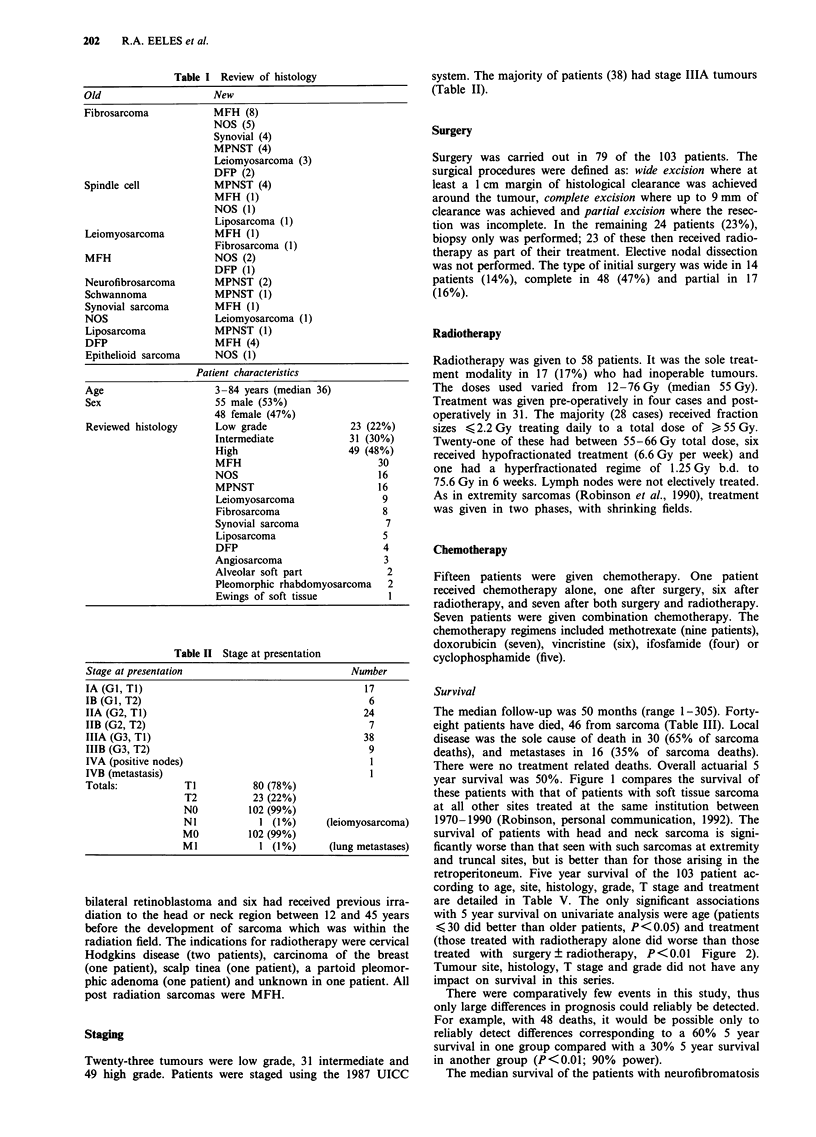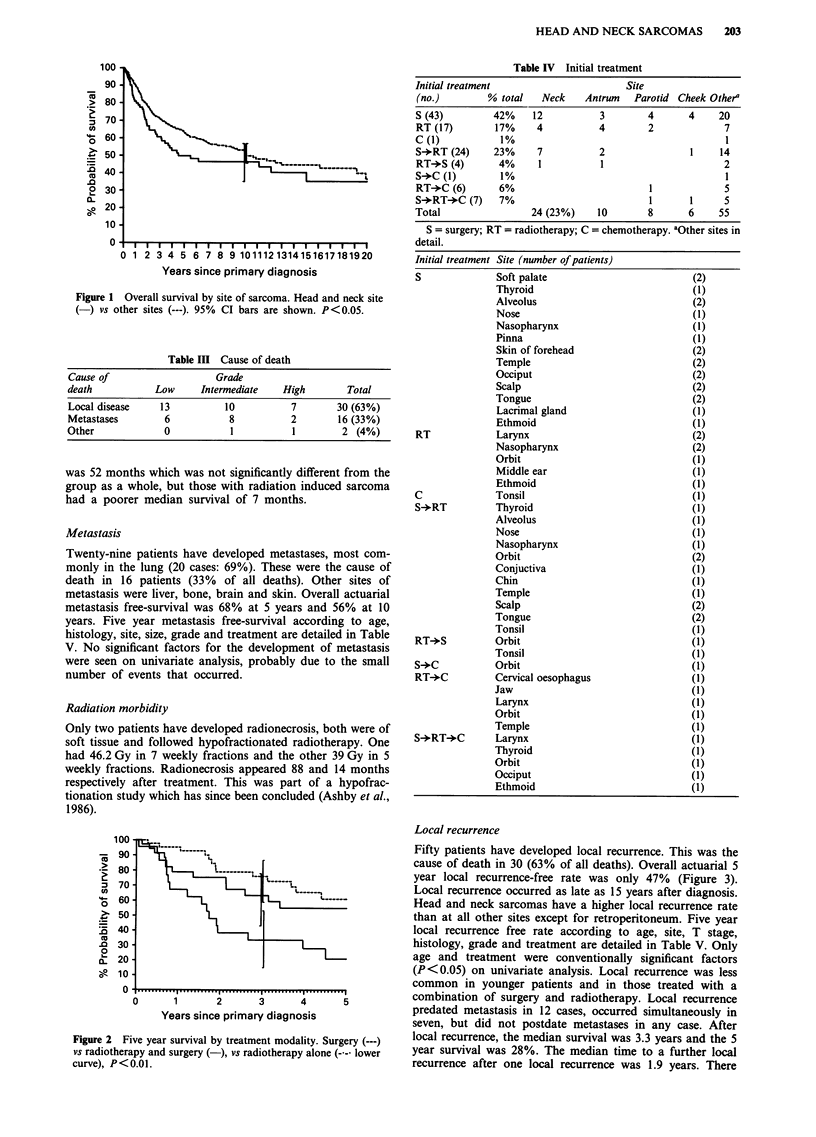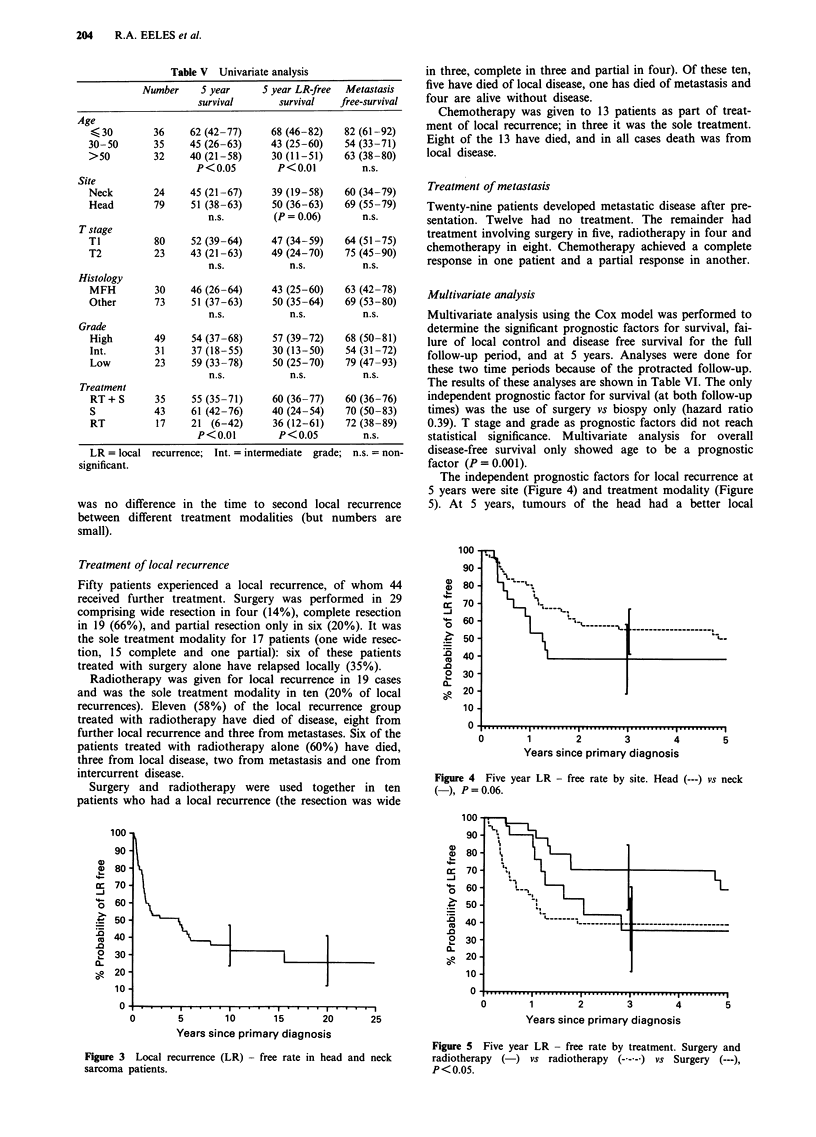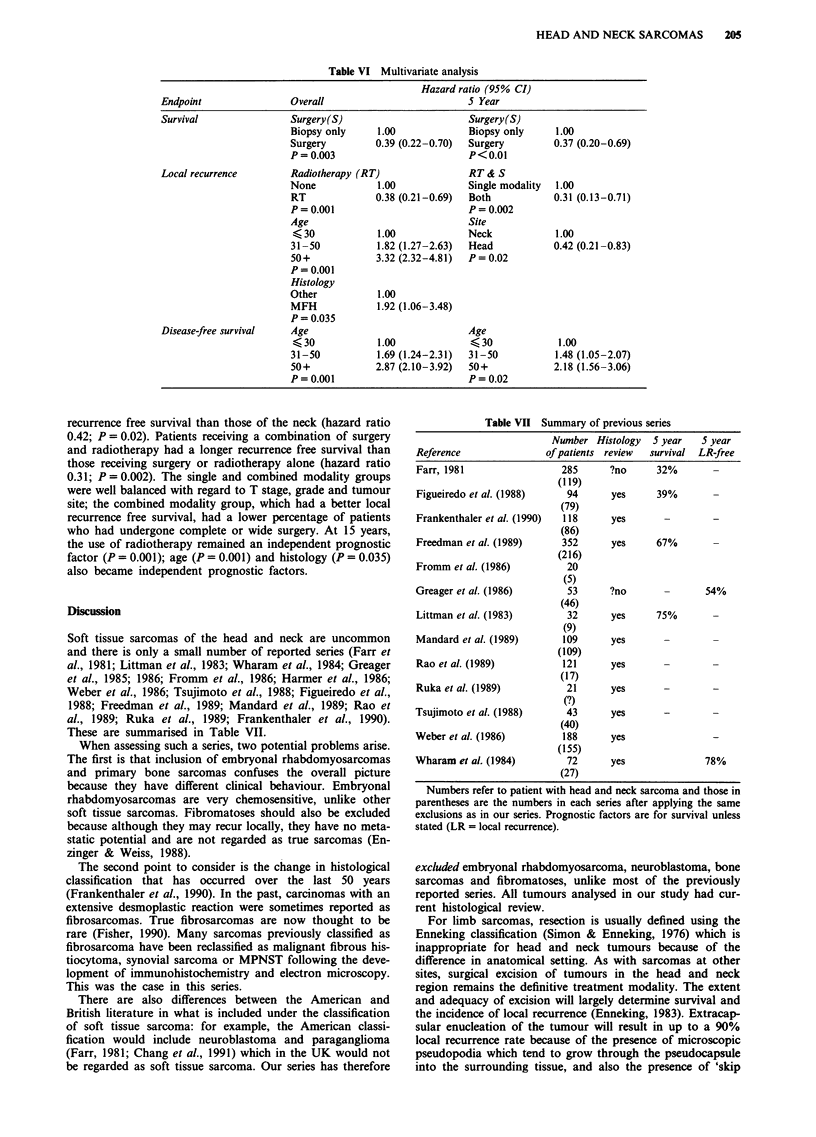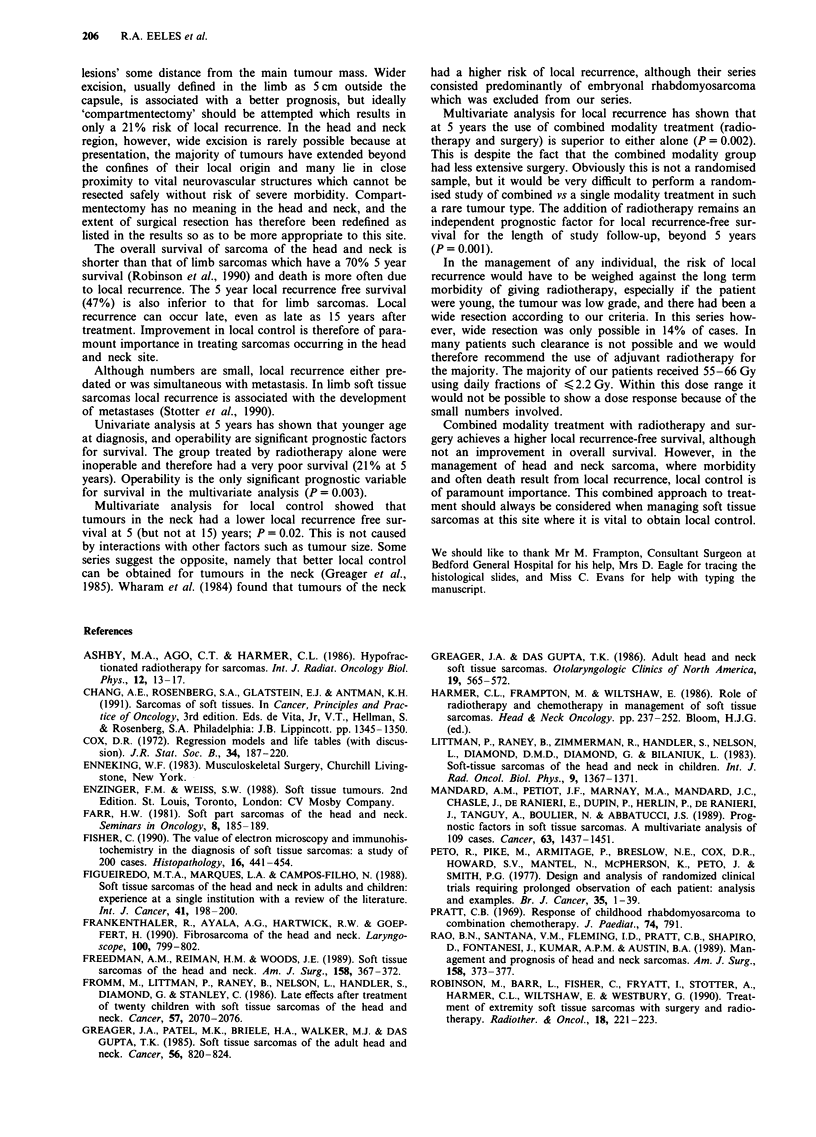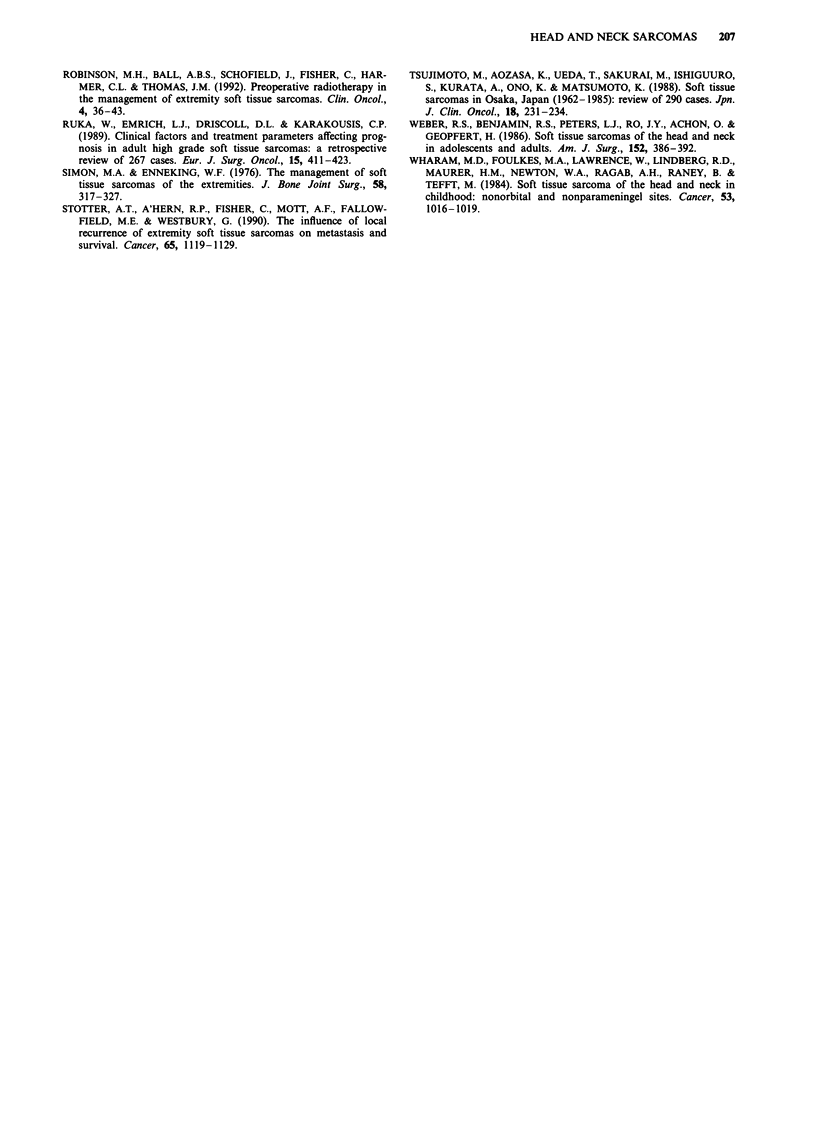# Head and neck sarcomas: prognostic factors and implications for treatment.

**DOI:** 10.1038/bjc.1993.314

**Published:** 1993-07

**Authors:** R. A. Eeles, C. Fisher, R. P. A'Hern, M. Robinson, P. Rhys-Evans, J. M. Henk, D. Archer, C. L. Harmer

**Affiliations:** Head and Neck Unit, Royal Marsden Hospital, London, UK.

## Abstract

One hundred and thirty patients with soft tissue sarcoma of the head and neck were treated at the Royal Marsden Hospital between 1944 and 1988. Pathological review was possible in 103 of these cases; only pathologically reviewed cases have been analysed. The median age at presentation was 36 years, and 53% were male. Four had neurofibromatosis type I, and one previous bilateral retinoblastoma. Six had undergone previous radiotherapy, 12 to 45 years prior to developing sarcoma. The tumours were < or = 5 cm in 78% of cases and high grade in 48%. Only one patient presented with lymph node metastases and only one with distant metastases (to lung). Malignant fibrous histiocytoma was the commonest histological type, occurring in 30 cases. The overall 5 year survival was 50% (95% CI 39-60). Local tumour was the cause of death in 63% of cases and 5 year local control was only 47% (95% CI 36-58) with local recurrence occurring as late as 15 years after treatment. The only favourable independent prognostic factor for survival was the ability to perform surgery (other than biopsy), with or without radiotherapy, as opposed to radiotherapy alone (hazard ratio 0.39; P = 0.003). Only one patient had a biopsy with no further treatment. Favourable independent prognostic factors for local control at 5 years were site (tumours of the head as opposed to the neck, hazard ratio 0.42; P = 0.02) and modality of treatment (combined surgery and radiotherapy compared to either alone, hazard ratio 0.31; P = 0.002). Patients in the combined modality and single treatment modality groups were well balanced for T stage, grade and tumour site. The patients in the combined treatment group had less extensive surgery, yet their local recurrence-free survival was longer. Unlike soft tissue sarcomas at other sites, those in the head and neck region more often cause death by local recurrence. The addition of radiotherapy to surgery may result in longer local recurrence-free survival.


Br. J. Cancer (1993), 68, 201–207 © Macmillan Press Ltd., 1993

### Head and neck sarcomas: prognostic factors and implications for treatment

R.A. Eeles[1,2]*, C. Fisher[2], R.P. A'Hern[3], M. Robinson[1], P. Rhys-Evans[1], J.M. Henk[1], D. Archer[1] & C.L. Harmer[2]

[1]*Head and Neck and* [2]*Sarcoma Units, and* [3]*Department of Computing, The Royal Marsden Hospital, Fulham Road, London SW3 6JJ, UK.*

**Summary** One hundred and thirty patients with soft tissue sarcoma of the head and neck were treated at the Royal Marsden Hospital between 1944 and 1988. Pathological review was possible in 103 of these cases; only pathologically reviewed cases have been analysed. The median age at presentation was 36 years, and 53% were male. Four had neurofibromatosis type I, and one previous bilateral retinoblastoma. Six had undergone previous radiotherapy, 12 to 45 years prior to developing sarcoma. The tumours were $\leqslant 5$ cm in 78% of cases and high grade in 48%. Only one patient presented with lymph node metastases and only one with distant metastases (to lung). Malignant fibrous histiocytoma was the commonest histological type, occurring in 30 cases.

The overall 5 year survival was 50% (95% CI 39–60). Local tumour was the cause of death in 63% of cases and 5 year local control was only 47% (95% CI 36–58) with local recurrence occurring as late as 15 years after treatment. The only favourable independent prognostic factor for survival was the ability to perform surgery (other than biopsy), with or without radiotherapy, as opposed to radiotherapy alone (hazard ratio 0.39; $P = 0.003$). Only one patient had a biopsy with no further treatment. Favourable independent prognostic factors for local control at 5 years were site (tumours of the head as opposed to the neck, hazard ratio 0.42; $P = 0.02$) and modality of treatment (combined surgery and radiotherapy compared to either alone, hazard ratio 0.31; $P = 0.002$). Patients in the combined modality and single treatment modality groups were well balanced for T stage, grade and tumour site. The patients in the combined treatment group had less extensive surgery, yet their local recurrence-free survival was longer.

Unlike soft tissue sarcomas at other sites, those in the head and neck region more often cause death by local recurrence. The addition of radiotherapy to surgery may result in longer local recurrence-free survival.

Soft tissue sarcomas of the head and neck are rare: they comprise <1% of head and neck cancers and <10% of all soft tissue sarcomas (Chang *et al.*, 1991). There have been few large series and most have included embryonal rhabdomyosarcomas which have a different natural history from other sarcomas and are both radio- and chemosensitive (Pratt, 1969). We have therefore carried out a retrospective study of all head and neck sarcomas treated at the Royal Marsden Hospital (RMH) from 1944–1988. Patients with embryonal rhabdomyosarcoma and fibromatoses were excluded. During the long time period involved, histological classification of these tumours had altered and so all the cases analysed were subjected to histological review. Prognostic factors for survival and local recurrence were studied. The implications for a combined surgical and radiotherapeutic approach to the treatment of these tumours are discussed.

### Patients

The medical records of patients referred to the RMH with a diagnosis of soft tissue sarcoma were reviewed. The head and neck was defined as any site above the clavicles. Fifty-eight patients with a diagnosis of embryonal rhabdomyosarcoma were excluded from this report because of the differences in clinical behaviour and resonse to treatment of this tumour type compared with other soft tissue sarcomas. Three patients with fibromatoses were also excluded because we do not consider this condition to be sarcomatous. The histological diagnoses were reviewed by one of us (CF), using additional staining procedures where appropriate. The histology review assigned the tumours into three grades (high, intermediate and low) using the following criteria: synovial sarcoma, epithelioid sarcoma, alveolar soft part sarcoma and undifferentiated sarcoma not otherwise specified (NOS) were always high grade. Well differentiated and myxoid liposarcoma were always low grade. Other tumour types were scored according to the extent of necrosis, cellularity, nuclear pleomorphism and mitotic activity, and graded according to the total score (Robinson *et al.*, 1992).

The following data were also collected: demographic factors, tumour stage (UICC), predisposing factors, treatment details, and local recurrence plus overall survival. Actuarial plots of survival and local recurrence-free rate, according to the above facors, were compared using log-rank analysis (Peto *et al.*, 1977). Multivariate Cox regression analysis was carried out to determine the independent prognostic factors for survival and local recurrence (Cox, 1972). Death was not treated as an event when analysing local recurrence-free rate.

### Results

Over 2,500 patients with sarcoma have been treated at the RMH since 1944. Of these, 130 had soft tissue sarcoma of the head and neck (with the exclusions mentioned in the patients' section) which is only 5% of the total. Histological slides from 103 of these patients were available for review (Table I) and these patients form the basis of this report. The patient characteristics are listed in Tables I and II. The commonest histological diagnosis was malignant fibrous histiocytoma or MFH (30), followed by undifferentiated sarcoma or NOS (16) and malignant peripheral nerve sheath tumour or MPNST (16). On review, the histology of 49 patients was changed (Table I). This was particularly common where the original diagnosis was fibrosarcoma (26 cases) when the reviewed diagnosis became MFH in eight cases, NOS in five, synovial sarcoma in four, MPNST in four, leiomyosarcoma in three and dermatofibrosarcoma protuberans (DFP) in two. In contrast, the diagnosis of one patient who had previously been classified as having a leiomyosarcoma became fibrosarcoma on review.

Predisposing factors to the development of sarcoma were noted in 11 patients. Four had neurofibromatosis type I, one

Correspondence: R.A. Eeles.
*Present address: CRC Academic Unit of Radiotherapy, The Royal Marsden Hospital, Down's Road, Sutton, Surrey SM2 5PT, UK.
Received 13 October 1993; and in revised form 5 March 1993.

**Table I** Review of histology

| Old | New | |
|---|---|---|
| Fibrosarcoma | MFH (8) | |
| | NOS (5) | |
| | Synovial (4) | |
| | MPNST (4) | |
| | Leiomyosarcoma (3) | |
| | DFP (2) | |
| Spindle cell | MPNST (4) | |
| | MFH (1) | |
| | NOS (1) | |
| | Liposarcoma (1) | |
| Leiomyosarcoma | MFH (1) | |
| | Fibrosarcoma (1) | |
| MFH | NOS (2) | |
| | DFP (1) | |
| Neurofibrosarcoma | MPNST (2) | |
| Schwannoma | MPNST (1) | |
| Synovial sarcoma | MFH (1) | |
| NOS | Leiomyosarcoma (1) | |
| Liposarcoma | MPNST (1) | |
| DFP | MFH (4) | |
| Epithelioid sarcoma | NOS (1) | |
| | *Patient characteristics* | |
| Age | 3–84 years (median 36) | |
| Sex | 55 male (53%) | |
| | 48 female (47%) | |
| Reviewed histology | Low grade | 23 (22%) |
| | Intermediate | 31 (30%) |
| | High | 49 (48%) |
| | MFH | 30 |
| | NOS | 16 |
| | MPNST | 16 |
| | Leiomyosarcoma | 9 |
| | Fibrosarcoma | 8 |
| | Synovial sarcoma | 7 |
| | Liposarcoma | 5 |
| | DFP | 4 |
| | Angiosarcoma | 3 |
| | Alveolar soft part | 2 |
| | Pleomorphic rhabdomyosarcoma | 2 |
| | Ewings of soft tissue | 1 |

**Table II** Stage at presentation

| Stage at presentation | | | Number |
|---|---|---|---|
| IA (G1, T1) | | | 17 |
| IB (G1, T2) | | | 6 |
| IIA (G2, T1) | | | 24 |
| IIB (G2, T2) | | | 7 |
| IIIA (G3, T1) | | | 38 |
| IIIB (G3, T2) | | | 9 |
| IVA (positive nodes) | | | 1 |
| IVB (metastasis) | | | 1 |
| Totals: | T1 | 80 (78%) | |
| | T2 | 23 (22%) | |
| | N0 | 102 (99%) | |
| | N1 | 1 (1%) | (leiomyosarcoma) |
| | M0 | 102 (99%) | |
| | M1 | 1 (1%) | (lung metastases) |

bilateral retinoblastoma and six had received previous irradiation to the head or neck region between 12 and 45 years before the development of sarcoma which was within the radiation field. The indications for radiotherapy were cervical Hodgkins disease (two patients), carcinoma of the breast (one patient), scalp tinea (one patient), a partoid pleomorphic adenoma (one patient) and unknown in one patient. All post radiation sarcomas were MFH.

### Staging

Twenty-three tumours were low grade, 31 intermediate and 49 high grade. Patients were staged using the 1987 UICC system. The majority of patients (38) had stage IIIA tumours (Table II).

### Surgery

Surgery was carried out in 79 of the 103 patients. The surgical procedures were defined as: *wide excision* where at least a 1 cm margin of histological clearance was achieved around the tumour, *complete excision* where up to 9 mm of clearance was achieved and *partial excision* where the resection was incomplete. In the remaining 24 patients (23%), biopsy only was performed; 23 of these then received radiotherapy as part of their treatment. Elective nodal dissection was not performed. The type of initial surgery was wide in 14 patients (14%), complete in 48 (47%) and partial in 17 (16%).

### Radiotherapy

Radiotherapy was given to 58 patients. It was the sole treatment modality in 17 (17%) who had inoperable tumours. The doses used varied from 12–76 Gy (median 55 Gy). Treatment was given pre-operatively in four cases and post-operatively in 31. The majority (28 cases) received fraction sizes $\leqslant$2.2 Gy treating daily to a total dose of $\geqslant$55 Gy. Twenty-one of these had between 55–66 Gy total dose, six received hypofractionated treatment (6.6 Gy per week) and one had a hyperfractionated regime of 1.25 Gy b.d. to 75.6 Gy in 6 weeks. Lymph nodes were not electively treated. As in extremity sarcomas (Robinson *et al.*, 1990), treatment was given in two phases, with shrinking fields.

### Chemotherapy

Fifteen patients were given chemotherapy. One patient received chemotherapy alone, one after surgery, six after radiotherapy, and seven after both surgery and radiotherapy. Seven patients were given combination chemotherapy. The chemotherapy regimens included methotrexate (nine patients), doxorubicin (seven), vincristine (six), ifosfamide (four) or cyclophosphamide (five).

### *Survival*

The median follow-up was 50 months (range 1–305). Forty-eight patients have died, 46 from sarcoma (Table III). Local disease was the sole cause of death in 30 (65% of sarcoma deaths), and metastases in 16 (35% of sarcoma deaths). There were no treatment related deaths. Overall actuarial 5 year survival was 50%. Figure 1 compares the survival of these patients with that of patients with soft tissue sarcoma at all other sites treated at the same institution between 1970–1990 (Robinson, personal communication, 1992). The survival of patients with head and neck sarcoma is significantly worse than that seen with such sarcomas at extremity and truncal sites, but is better than for those arising in the retroperitoneum. Five year survival of the 103 patient according to age, site, histology, grade, T stage and treatment are detailed in Table V. The only significant associations with 5 year survival on univariate analysis were age (patients $\leqslant$30 did better than older patients, $P<0.05$) and treatment (those treated with radiotherapy alone did worse than those treated with surgery $\pm$ radiotherapy, $P<0.01$ Figure 2). Tumour site, histology, T stage and grade did not have any impact on survival in this series.

There were comparatively few events in this study, thus only large differences in prognosis could reliably be detected. For example, with 48 deaths, it would be possible only to reliably detect differences corresponding to a 60% 5 year survival in one group compared with a 30% 5 year survival in another group ($P<0.01$; 90% power).

The median survival of the patients with neurofibromatosis

**Figure 1** Overall survival by site of sarcoma. Head and neck site (—) *vs* other sites (---). 95% CI bars are shown. $P<0.05$.

**Table III** Cause of death

| *Cause of death* | *Grade* Low | Intermediate | High | Total |
|---|---|---|---|---|
| Local disease | 13 | 10 | 7 | 30 (63%) |
| Metastases | 6 | 8 | 2 | 16 (33%) |
| Other | 0 | 1 | 1 | 2 (4%) |

was 52 months which was not significantly different from the group as a whole, but those with radiation induced sarcoma had a poorer median survival of 7 months.

### *Metastasis*

Twenty-nine patients have developed metastases, most commonly in the lung (20 cases: 69%). These were the cause of death in 16 patients (33% of all deaths). Other sites of metastasis were liver, bone, brain and skin. Overall actuarial metastasis free-survival was 68% at 5 years and 56% at 10 years. Five year metastasis free-survival according to age, histology, site, size, grade and treatment are detailed in Table V. No significant factors for the development of metastasis were seen on univariate analysis, probably due to the small number of events that occurred.

### *Radiation morbidity*

Only two patients have developed radionecrosis, both were of soft tissue and followed hypofractionated radiotherapy. One had 46.2 Gy in 7 weekly fractions and the other 39 Gy in 5 weekly fractions. Radionecrosis appeared 88 and 14 months respectively after treatment. This was part of a hypofractionation study which has since been concluded (Ashby *et al*., 1986).

**Figure 2** Five year survival by treatment modality. Surgery (---) *vs* radiotherapy and surgery (—), *vs* radiotherapy alone (-·-- lower curve), $P<0.01$.

**Table IV** Initial treatment

| *Initial treatment (no.)* | *% total* | *Site* Neck | Antrum | Parotid | Cheek | Other[a] |
|---|---|---|---|---|---|---|
| S (43) | 42% | 12 | 3 | 4 | 4 | 20 |
| RT (17) | 17% | 4 | 4 | 2 | | 7 |
| C (1) | 1% | | | | | 1 |
| S→RT (24) | 23% | 7 | 2 | | 1 | 14 |
| RT→S (4) | 4% | 1 | 1 | | | 2 |
| S→C (1) | 1% | | | | | 1 |
| RT→C (6) | 6% | | | 1 | | 5 |
| S→RT→C (7) | 7% | | | 1 | 1 | 5 |
| Total | | 24 (23%) | 10 | 8 | 6 | 55 |

S = surgery; RT = radiotherapy; C = chemotherapy. [a]Other sites in detail.

| *Initial treatment* | *Site (number of patients)* | |
|---|---|---|
| S | Soft palate | (2) |
| | Thyroid | (1) |
| | Alveolus | (2) |
| | Nose | (1) |
| | Nasopharynx | (1) |
| | Pinna | (1) |
| | Skin of forehead | (2) |
| | Temple | (2) |
| | Occiput | (2) |
| | Scalp | (2) |
| | Tongue | (2) |
| | Lacrimal gland | (1) |
| | Ethmoid | (1) |
| RT | Larynx | (2) |
| | Nasopharynx | (2) |
| | Orbit | (1) |
| | Middle ear | (1) |
| | Ethmoid | (1) |
| C | Tonsil | (1) |
| S→RT | Thyroid | (1) |
| | Alveolus | (1) |
| | Nose | (1) |
| | Nasopharynx | (1) |
| | Orbit | (2) |
| | Conjuctiva | (1) |
| | Chin | (1) |
| | Temple | (1) |
| | Scalp | (2) |
| | Tongue | (2) |
| | Tonsil | (1) |
| RT→S | Orbit | (1) |
| | Tonsil | (1) |
| S→C | Orbit | (1) |
| RT→C | Cervical oesophagus | (1) |
| | Jaw | (1) |
| | Larynx | (1) |
| | Orbit | (1) |
| | Temple | (1) |
| S→RT→C | Larynx | (1) |
| | Thyroid | (1) |
| | Orbit | (1) |
| | Occiput | (1) |
| | Ethmoid | (1) |

### *Local recurrence*

Fifty patients have developed local recurrence. This was the cause of death in 30 (63% of all deaths). Overall actuarial 5 year local recurrence-free rate was only 47% (Figure 3). Local recurrence occurred as late as 15 years after diagnosis. Head and neck sarcomas have a higher local recurrence rate than at all other sites except for retroperitoneum. Five year local recurrence free rate according to age, site, T stage, histology, grade and treatment are detailed in Table V. Only age and treatment were conventionally significant factors ($P<0.05$) on univariate analysis. Local recurrence was less common in younger patients and in those treated with a combination of surgery and radiotherapy. Local recurrence predated metastasis in 12 cases, occurred simultaneously in seven, but did not postdate metastases in any case. After local recurrence, the median survival was 3.3 years and the 5 year survival was 28%. The median time to a further local recurrence after one local recurrence was 1.9 years. There

**Table V** Univariate analysis

| | Number | 5 year survival | 5 year LR-free survival | Metastasis free-survival |
|---|---|---|---|---|
| *Age* | | | | |
| ⩽30 | 36 | 62 (42–77) | 68 (46–82) | 82 (61–92) |
| 30–50 | 35 | 45 (26–63) | 43 (25–60) | 54 (33–71) |
| >50 | 32 | 40 (21–58) | 30 (11–51) | 63 (38–80) |
| | | $P<0.05$ | $P<0.01$ | n.s. |
| *Site* | | | | |
| Neck | 24 | 45 (21–67) | 39 (19–58) | 60 (34–79) |
| Head | 79 | 51 (38–63) | 50 (36–63) | 69 (55–79) |
| | | n.s. | ($P=0.06$) | n.s. |
| *T stage* | | | | |
| T1 | 80 | 52 (39–64) | 47 (34–59) | 64 (51–75) |
| T2 | 23 | 43 (21–63) | 49 (24–70) | 75 (45–90) |
| | | n.s. | n.s. | n.s. |
| *Histology* | | | | |
| MFH | 30 | 46 (26–64) | 43 (25–60) | 63 (42–78) |
| Other | 73 | 51 (37–63) | 50 (35–64) | 69 (53–80) |
| | | n.s. | n.s. | n.s. |
| *Grade* | | | | |
| High | 49 | 54 (37–68) | 57 (39–72) | 68 (50–81) |
| Int. | 31 | 37 (18–55) | 30 (13–50) | 54 (31–72) |
| Low | 23 | 59 (33–78) | 50 (25–70) | 79 (47–93) |
| | | n.s. | n.s. | n.s. |
| *Treatment* | | | | |
| RT + S | 35 | 55 (35–71) | 60 (36–77) | 60 (36–76) |
| S | 43 | 61 (42–76) | 40 (24–54) | 70 (50–83) |
| RT | 17 | 21 (6–42) | 36 (12–61) | 72 (38–89) |
| | | $P<0.01$ | $P<0.05$ | n.s. |

LR = local recurrence; Int. = intermediate grade; n.s. = non-significant.

was no difference in the time to second local recurrence between different treatment modalities (but numbers are small).

### *Treatment of local recurrence*

Fifty patients experienced a local recurrence, of whom 44 received further treatment. Surgery was performed in 29 comprising wide resection in four (14%), complete resection in 19 (66%), and partial resection only in six (20%). It was the sole treatment modality for 17 patients (one wide resection, 15 complete and one partial): six of these patients treated with surgery alone have relapsed locally (35%).

Radiotherapy was given for local recurrence in 19 cases and was the sole treatment modality in ten (20% of local recurrences). Eleven (58%) of the local recurrence group treated with radiotherapy have died of disease, eight from further local recurrence and three from metastases. Six of the patients treated with radiotherapy alone (60%) have died, three from local disease, two from metastasis and one from intercurrent disease.

Surgery and radiotherapy were used together in ten patients who had a local recurrence (the resection was wide in three, complete in three and partial in four). Of these ten, five have died of local disease, one has died of metastasis and four are alive without disease.

Chemotherapy was given to 13 patients as part of treatment of local recurrence; in three it was the sole treatment. Eight of the 13 have died, and in all cases death was from local disease.

### *Treatment of metastasis*

Twenty-nine patients developed metastatic disease after presentation. Twelve had no treatment. The remainder had treatment involving surgery in five, radiotherapy in four and chemotherapy in eight. Chemotherapy achieved a complete response in one patient and a partial response in another.

### *Multivariate analysis*

Multivariate analysis using the Cox model was performed to determine the significant prognostic factors for survival, failure of local control and disease free survival for the full follow-up period, and at 5 years. Analyses were done for these two time periods because of the protracted follow-up. The results of these analyses are shown in Table VI. The only independent prognostic factor for survival (at both follow-up times) was the use of surgery *vs* biospy only (hazard ratio 0.39). T stage and grade as prognostic factors did not reach statistical significance. Multivariate analysis for overall disease-free survival only showed age to be a prognostic factor ($P=0.001$).

The independent prognostic factors for local recurrence at 5 years were site (Figure 4) and treatment modality (Figure 5). At 5 years, tumours of the head had a better local

**Figure 3** Local recurrence (LR) – free rate in head and neck sarcoma patients.

**Figure 4** Five year LR – free rate by site. Head (---) *vs* neck (—), $P=0.06$.

**Figure 5** Five year LR – free rate by treatment. Surgery and radiotherapy (—) *vs* radiotherapy (-·-·-) *vs* Surgery (---), $P<0.05$.

**Table VI** Multivariate analysis

| Endpoint | Hazard ratio (95% CI) Overall | | 5 Year | |
|---|---|---|---|---|
| *Survival* | *Surgery(S)* | | *Surgery(S)* | |
| | Biopsy only | 1.00 | Biopsy only | 1.00 |
| | Surgery | 0.39 (0.22–0.70) | Surgery | 0.37 (0.20–0.69) |
| | $P = 0.003$ | | $P < 0.01$ | |
| *Local recurrence* | *Radiotherapy (RT)* | | *RT & S* | |
| | None | 1.00 | Single modality | 1.00 |
| | RT | 0.38 (0.21–0.69) | Both | 0.31 (0.13–0.71) |
| | $P = 0.001$ | | $P = 0.002$ | |
| | *Age* | | *Site* | |
| | ⩽30 | 1.00 | Neck | 1.00 |
| | 31–50 | 1.82 (1.27–2.63) | Head | 0.42 (0.21–0.83) |
| | 50+ | 3.32 (2.32–4.81) | $P = 0.02$ | |
| | $P = 0.001$ | | | |
| | *Histology* | | | |
| | Other | 1.00 | | |
| | MFH | 1.92 (1.06–3.48) | | |
| | $P = 0.035$ | | | |
| *Disease-free survival* | *Age* | | *Age* | |
| | ⩽30 | 1.00 | ⩽30 | 1.00 |
| | 31–50 | 1.69 (1.24–2.31) | 31–50 | 1.48 (1.05–2.07) |
| | 50+ | 2.87 (2.10–3.92) | 50+ | 2.18 (1.56–3.06) |
| | $P = 0.001$ | | $P = 0.02$ | |

recurrence free survival than those of the neck (hazard ratio 0.42; $P = 0.02$). Patients receiving a combination of surgery and radiotherapy had a longer recurrence free survival than those receiving surgery or radiotherapy alone (hazard ratio 0.31; $P = 0.002$). The single and combined modality groups were well balanced with regard to T stage, grade and tumour site; the combined modality group, which had a better local recurrence free survival, had a lower percentage of patients who had undergone complete or wide surgery. At 15 years, the use of radiotherapy remained an independent prognostic factor ($P = 0.001$); age ($P = 0.001$) and histology ($P = 0.035$) also became independent prognostic factors.

### Discussion

Soft tissue sarcomas of the head and neck are uncommon and there is only a small number of reported series (Farr *et al.*, 1981; Littman *et al.*, 1983; Wharam *et al.*, 1984; Greager *et al.*, 1985; 1986; Fromm *et al.*, 1986; Harmer *et al.*, 1986; Weber *et al.*, 1986; Tsujimoto *et al.*, 1988; Figueiredo *et al.*, 1988; Freedman *et al.*, 1989; Mandard *et al.*, 1989; Rao *et al.*, 1989; Ruka *et al.*, 1989; Frankenthaler *et al.*, 1990). These are summarised in Table VII.

When assessing such a series, two potential problems arise. The first is that inclusion of embryonal rhabdomyosarcomas and primary bone sarcomas confuses the overall picture because they have different clinical behaviour. Embryonal rhabdomyosarcomas are very chemosensitive, unlike other soft tissue sarcomas. Fibromatoses should also be excluded because although they may recur locally, they have no metastatic potential and are not regarded as true sarcomas (Enzinger & Weiss, 1988).

The second point to consider is the change in histological classification that has occurred over the last 50 years (Frankenthaler *et al.*, 1990). In the past, carcinomas with an extensive desmoplastic reaction were sometimes reported as fibrosarcomas. True fibrosarcomas are now thought to be rare (Fisher, 1990). Many sarcomas previously classified as fibrosarcoma have been reclassified as malignant fibrous histiocytoma, synovial sarcoma or MPNST following the development of immunohistochemistry and electron microscopy. This was the case in this series.

There are also differences between the American and British literature in what is included under the classification of soft tissue sarcoma: for example, the American classification would include neuroblastoma and paraganglioma (Farr, 1981; Chang *et al.*, 1991) which in the UK would not be regarded as soft tissue sarcoma. Our series has therefore

**Table VII** Summary of previous series

| Reference | Number of patients | Histology review | 5 year survival | 5 year LR-free |
|---|---|---|---|---|
| Farr, 1981 | 285 (119) | ?no | 32% | – |
| Figueiredo *et al.* (1988) | 94 (79) | yes | 39% | – |
| Frankenthaler *et al.* (1990) | 118 (86) | yes | – | – |
| Freedman *et al.* (1989) | 352 (216) | yes | 67% | – |
| Fromm *et al.* (1986) | 20 (5) | | | |
| Greager *et al.* (1986) | 53 (46) | ?no | – | 54% |
| Littman *et al.* (1983) | 32 (9) | yes | 75% | – |
| Mandard *et al.* (1989) | 109 (109) | yes | – | – |
| Rao *et al.* (1989) | 121 (17) | yes | – | – |
| Ruka *et al.* (1989) | 21 (?) | yes | – | – |
| Tsujimoto *et al.* (1988) | 43 (40) | yes | – | – |
| Weber *et al.* (1986) | 188 (155) | yes | | – |
| Wharam *et al.* (1984) | 72 (27) | yes | | 78% |

Numbers refer to patient with head and neck sarcoma and those in parentheses are the numbers in each series after applying the same exclusions as in our series. Prognostic factors are for survival unless stated (LR = local recurrence).

*excluded* embryonal rhabdomyosarcoma, neuroblastoma, bone sarcomas and fibromatoses, unlike most of the previously reported series. All tumours analysed in our study had current histological review.

For limb sarcomas, resection is usually defined using the Enneking classification (Simon & Enneking, 1976) which is inappropriate for head and neck tumours because of the difference in anatomical setting. As with sarcomas at other sites, surgical excision of tumours in the head and neck region remains the definitive treatment modality. The extent and adequacy of excision will largely determine survival and the incidence of local recurrence (Enneking, 1983). Extracapsular enucleation of the tumour will result in up to a 90% local recurrence rate because of the presence of microscopic pseudopodia which tend to grow through the pseudocapsule into the surrounding tissue, and also the presence of 'skip lesions' some distance from the main tumour mass. Wider excision, usually defined in the limb as 5 cm outside the capsule, is associated with a better prognosis, but ideally 'compartmentectomy' should be attempted which results in only a 21% risk of local recurrence. In the head and neck region, however, wide excision is rarely possible because at presentation, the majority of tumours have extended beyond the confines of their local origin and many lie in close proximity to vital neurovascular structures which cannot be resected safely without risk of severe morbidity. Compartmentectomy has no meaning in the head and neck, and the extent of surgical resection has therefore been redefined as listed in the results so as to be more appropriate to this site.

The overall survival of sarcoma of the head and neck is shorter than that of limb sarcomas which have a 70% 5 year survival (Robinson *et al.*, 1990) and death is more often due to local recurrence. The 5 year local recurrence free survival (47%) is also inferior to that for limb sarcomas. Local recurrence can occur late, even as late as 15 years after treatment. Improvement in local control is therefore of paramount importance in treating sarcomas occurring in the head and neck site.

Although numbers are small, local recurrence either predated or was simultaneous with metastasis. In limb soft tissue sarcomas local recurrence is associated with the development of metastases (Stotter *et al.*, 1990).

Univariate analysis at 5 years has shown that younger age at diagnosis, and operability are significant prognostic factors for survival. The group treated by radiotherapy alone were inoperable and therefore had a very poor survival (21% at 5 years). Operability is the only significant prognostic variable for survival in the multivariate analysis ($P = 0.003$).

Multivariate analysis for local control showed that tumours in the neck had a lower local recurrence free survival at 5 (but not at 15) years; $P = 0.02$. This is not caused by interactions with other factors such as tumour size. Some series suggest the opposite, namely that better local control can be obtained for tumours in the neck (Greager *et al.*, 1985). Wharam *et al.* (1984) found that tumours of the neck had a higher risk of local recurrence, although their series consisted predominantly of embryonal rhabdomyosarcoma which was excluded from our series.

Multivariate analysis for local recurrence has shown that at 5 years the use of combined modality treatment (radiotherapy and surgery) is superior to either alone ($P = 0.002$). This is despite the fact that the combined modality group had less extensive surgery. Obviously this is not a randomised sample, but it would be very difficult to perform a randomised study of combined *vs* a single modality treatment in such a rare tumour type. The addition of radiotherapy remains an independent prognostic factor for local recurrence-free survival for the length of study follow-up, beyond 5 years ($P = 0.001$).

In the management of any individual, the risk of local recurrence would have to be weighed against the long term morbidity of giving radiotherapy, especially if the patient were young, the tumour was low grade, and there had been a wide resection according to our criteria. In this series however, wide resection was only possible in 14% of cases. In many patients such clearance is not possible and we would therefore recommend the use of adjuvant radiotherapy for the majority. The majority of our patients received 55–66 Gy using daily fractions of $\leqslant$2.2 Gy. Within this dose range it would not be possible to show a dose response because of the small numbers involved.

Combined modality treatment with radiotherapy and surgery achieves a higher local recurrence-free survival, although not an improvement in overall survival. However, in the management of head and neck sarcoma, where morbidity and often death result from local recurrence, local control is of paramount importance. This combined approach to treatment should always be considered when managing soft tissue sarcomas at this site where it is vital to obtain local control.

We should like to thank Mr M. Frampton, Consultant Surgeon at Bedford General Hospital for his help, Mrs D. Eagle for tracing the histological slides, and Miss C. Evans for help with typing the manuscript.

### References

ASHBY, M.A., AGO, C.T. & HARMER, C.L. (1986). Hypofractionated radiotherapy for sarcomas. *Int. J. Radiat. Oncology Biol. Phys.*, **12**, 13–17.

CHANG, A.E., ROSENBERG, S.A., GLATSTEIN, E.J. & ANTMAN, K.H. (1991). Sarcomas of soft tissues. In *Cancer, Principles and Practice of Oncology*, 3rd edition. Eds. de Vita, Jr, V.T., Hellman, S. & Rosenberg, S.A. Philadelphia: J.B. Lippincott. pp. 1345–1350.

COX, D.R. (1972). Regression models and life tables (with discussion). *J.R. Stat. Soc. B.*, **34**, 187–220.

ENNEKING, W.F. (1983). Musculoskeletal Surgery, Churchill Livingstone, New York.

ENZINGER, F.M. & WEISS, S.W. (1988). Soft tissue tumours. 2nd Edition. St. Louis, Toronto, London: CV Mosby Company.

FARR, H.W. (1981). Soft part sarcomas of the head and neck. *Seminars in Oncology*, **8**, 185–189.

FISHER, C. (1990). The value of electron microscopy and immunohistochemistry in the diagnosis of soft tissue sarcomas: a study of 200 cases. *Histopathology*, **16**, 441–454.

FIGUEIREDO, M.T.A., MARQUES, L.A. & CAMPOS-FILHO, N. (1988). Soft tissue sarcomas of the head and neck in adults and children: experience at a single institution with a review of the literature. *Int. J. Cancer*, **41**, 198–200.

FRANKENTHALER, R., AYALA, A.G., HARTWICK, R.W. & GOEPFERT, H. (1990). Fibrosarcoma of the head and neck. *Laryngoscope*, **100**, 799–802.

FREEDMAN, A.M., REIMAN, H.M. & WOODS, J.E. (1989). Soft tissue sarcomas of the head and neck. *Am. J. Surg.*, **158**, 367–372.

FROMM, M., LITTMAN, P., RANEY, B., NELSON, L., HANDLER, S., DIAMOND, G. & STANLEY, C. (1986). Late effects after treatment of twenty children with soft tissue sarcomas of the head and neck. *Cancer*, **57**, 2070–2076.

GREAGER, J.A., PATEL, M.K., BRIELE, H.A., WALKER, M.J. & DAS GUPTA, T.K. (1985). Soft tissue sarcomas of the adult head and neck. *Cancer*, **56**, 820–824.

GREAGER, J.A. & DAS GUPTA, T.K. (1986). Adult head and neck soft tissue sarcomas. *Otolaryngologic Clinics of North America*, **19**, 565–572.

HARMER, C.L., FRAMPTON, M. & WILTSHAW, E. (1986). Role of radiotherapy and chemotherapy in management of soft tissue sarcomas. *Head & Neck Oncology*. pp. 237–252. Bloom, H.J.G. (ed.).

LITTMAN, P., RANEY, B., ZIMMERMAN, R., HANDLER, S., NELSON, L., DIAMOND, D.M.D., DIAMOND, G. & BILANIUK, L. (1983). Soft-tissue sarcomas of the head and neck in children. *Int. J. Rad. Oncol. Biol. Phys.*, **9**, 1367–1371.

MANDARD, A.M., PETIOT, J.F., MARNAY, M.A., MANDARD, J.C., CHASLE, J., DE RANIERI, E., DUPIN, P., HERLIN, P., DE RANIERI, J., TANGUY, A., BOULIER, N. & ABBATUCCI, J.S. (1989). Prognostic factors in soft tissue sarcomas. A multivariate analysis of 109 cases. *Cancer*, **63**, 1437–1451.

PETO, R., PIKE, M., ARMITAGE, P., BRESLOW, N.E., COX, D.R., HOWARD, S.V., MANTEL, N., MCPHERSON, K., PETO, J. & SMITH, P.G. (1977). Design and analysis of randomized clinical trials requiring prolonged observation of each patient: analysis and examples. *Br. J. Cancer*, **35**, 1–39.

PRATT, C.B. (1969). Response of childhood rhabdomyosarcoma to combination chemotherapy. *J. Paediat.*, **74**, 791.

RAO, B.N., SANTANA, V.M., FLEMING, I.D., PRATT, C.B., SHAPIRO, D., FONTANESI, J., KUMAR, A.P.M. & AUSTIN, B.A. (1989). Management and prognosis of head and neck sarcomas. *Am. J. Surg.*, **158**, 373–377.

ROBINSON, M., BARR, L., FISHER, C., FRYATT, I., STOTTER, A., HARMER, C.L., WILTSHAW, E. & WESTBURY, G. (1990). Treatment of extremity soft tissue sarcomas with surgery and radiotherapy. *Radiother. & Oncol.*, **18**, 221–223.

ROBINSON, M.H., BALL, A.B.S., SCHOFIELD, J., FISHER, C., HARMER, C.L. & THOMAS, J.M. (1992). Preoperative radiotherapy in the management of extremity soft tissue sarcomas. *Clin. Oncol.*, **4**, 36–43.

RUKA, W., EMRICH, L.J., DRISCOLL, D.L. & KARAKOUSIS, C.P. (1989). Clinical factors and treatment parameters affecting prognosis in adult high grade soft tissue sarcomas: a retrospective review of 267 cases. *Eur. J. Surg. Oncol.*, **15**, 411–423.

SIMON, M.A. & ENNEKING, W.F. (1976). The management of soft tissue sarcomas of the extremities. *J. Bone Joint Surg.*, **58**, 317–327.

STOTTER, A.T., A'HERN, R.P., FISHER, C., MOTT, A.F., FALLOWFIELD, M.E. & WESTBURY, G. (1990). The influence of local recurrence of extremity soft tissue sarcomas on metastasis and survival. *Cancer*, **65**, 1119–1129.

TSUJIMOTO, M., AOZASA, K., UEDA, T., SAKURAI, M., ISHIGUURO, S., KURATA, A., ONO, K. & MATSUMOTO, K. (1988). Soft tissue sarcomas in Osaka, Japan (1962–1985): review of 290 cases. *Jpn. J. Clin. Oncol.*, **18**, 231–234.

WEBER, R.S., BENJAMIN, R.S., PETERS, L.J., RO, J.Y., ACHON, O. & GEOPFERT, H. (1986). Soft tissue sarcomas of the head and neck in adolescents and adults. *Am. J. Surg.*, **152**, 386–392.

WHARAM, M.D., FOULKES, M.A., LAWRENCE, W., LINDBERG, R.D., MAURER, H.M., NEWTON, W.A., RAGAB, A.H., RANEY, B. & TEFFT, M. (1984). Soft tissue sarcoma of the head and neck in childhood: nonorbital and nonparameningel sites. *Cancer*, **53**, 1016–1019.